# What do young adults know about the HIV/AIDS epidemic? Findings from a population based study in Karachi, Pakistan

**DOI:** 10.1186/1471-2334-9-38

**Published:** 2009-03-26

**Authors:** Syed Farid-ul-Hasnain, Eva Johansson, Gunilla Krantz

**Affiliations:** 1 Department of Community Health Sciences, Aga Khan University, Karachi, Pakistan; 2 Department of Public Health Sciences, Division of Global Health, IHCAR, Karolinska Institutet, Stockholm, Sweden; 3 Nordic School of Public Health, Gothenburg, Sweden; 4 Dept of Community Medicine and Public Health, The Sahlgrenska Academy at University of Gothenburg, Gothenburg, Sweden

## Abstract

**Background:**

HIVAIDS is spreading globally, hitting the younger generations. In Pakistan, the prevalence of HIV in high-risk subpopulations is five per cent or higher. This poses a serious threat of a generalised epidemic especially among the younger population. In the wake of HIVAIDS epidemic this is worrying as a well informed younger generation is crucial in restricting the spread of this epidemic. This study investigated Pakistani young adults' (male and female) knowledge and awareness of the HIV/AIDS disease.

**Methods:**

A population-based, cross-sectional study of 1,650 male and female adults aged 17–21 years living in Karachi was conducted using a structured questionnaire. A multi-stage cluster sampling design was used to collect data representative of the general population in an urban area. Bivariate and multivariate analyses were performed separately for males and females.

**Results:**

Of 1,650 subjects, 24 per cent (n = 390) reported that they had not heard of HIV/AIDS. Among the males, those with a poor knowledge were younger (AOR = 2.20; 95 per cent CI, 1.38, 3.49), with less than six years of schooling (AOR = 2.46; 1.29 4.68) and no computer at home (AOR = 1.88; 1.06 3.34). Among the females, the risk factors for poor knowledge were young age (AOR = 1.74; 1.22, 2.50), low socio-economic status (AOR = 1.54; 1.06, 2.22), lack of enrolment at school/college (AOR = 1.61; 1.09, 2.39) and being unmarried (AOR = 1.85; 1.05, 3.26).

**Conclusion:**

Alarming gaps in knowledge relating to HIV/AIDS were detected. The study emphasises the need to educate young adults and equip them with the appropriate information and skills to enable them to protect themselves from HIV/AIDS. However, taboos surrounding public discussions of sexuality remain a key constraint to preventive activities.

## Background

Young people aged 15–24 account for an estimated 45% of new HIV infections worldwide [1]. Even though the present prevalence of HIV is comparatively low in many Asian countries, epidemics in Indonesia, Pakistan, and Vietnam are growing rapidly [1]. Alarming gaps in knowledge relating to the HIV/AIDS epidemic among young adults are frequently reported [2,3].

According to UNAIDS estimates, the prevalence of HIV/AIDS among the population of men and women aged 15–24 in Pakistan is about 0.1 per cent [1]. Surveillance data from the Sindh provincial AIDS control programme indicate that the country has, however entered the "concentrated epidemic" stage for HIV/AIDS, in the sense that the HIV prevalence in high-risk subpopulations is five per cent or higher [4,5]. These high-risk subpopulations are injecting drug user communities and commercial sex networks in larger cities in Pakistan [6,7]. This poses a serious threat of a generalised epidemic, especially among the younger population [4,5].

Between 1981 and 2000, the mean age at marriage in Pakistan rose from 25.1 to 26.3 years for males and 20.2 to 22.1 years for females [8]. This indicates a widening window during which young people are likely to engage in risky sexual behaviour. The estimated population of adolescents (10–19 years of age) in Pakistan is over 30 million, which is approximately 23 per cent of the total population [9].

Youth populations are likely to engage in high-risk sexual behaviour. In those low-income countries where males are assigned a higher social status than females, it is mostly put on young women's shoulders to protect themselves from sexually transmitted infections and unwanted pregnancies but at the same time they often lack access to appropriate information about contraceptives and have poor access to relevant services for prevention and treatment [10-12].

Access and exposure to appropriate HIV/AIDS information and discussing it with others has the potential positively to impact knowledge, attitudes, beliefs and sexual practices [13]. Still, youth populations are poorly informed in Pakistan due to limited access to information about sexual and reproductive health matters, as both parents and the school system are reluctant to fulfil this obligation due to the sensitivity of the subject [14,15]. On the other hand, younger population obtain this information through the media, such as TV and the internet [16], but relying on such sources of information for the nation's younger generations is not advisable. Considering this, and the fact that the HIV/AIDS epidemic is on the increase in Pakistan makes it extremely important to assess young people's knowledge and perceptions of sexual and reproductive health matters and of the HIV/AIDS epidemic in particular to identify knowledge gaps for further preventive activities.

This population-based study assessed the level of knowledge and awareness of the HIV/AIDS epidemic among young adults, aged 17–21 years, in Karachi, Pakistan, focusing on modes of spread and preventive measures. Males and females were compared and particular risk groups were identified.

## Methods

### Population

This population-based, cross-sectional study of young adults aged 17–21 years was conducted in the metropolitan city of Karachi, where the population consists of several ethnic groups from all areas of the country. The sample size was calculated at a total of 1,650 with the assumption that 50 and 57 per cent of the males and females respectively would have insufficient knowledge of the modes of spread of HIV/AIDS [17,18], applying a 95 per cent confidence level, a power of 80 per cent and including an equal number of males and females. The sample size was also calculated while incorporating the element of design effect. The data collection was carried out between June and August 2006.

### Sampling

A multi-stage cluster sampling design was used to identify the respondents. The city of Karachi comprises 18 towns (administrative units), further divided into union councils. The calculated sample size (1,650) was then divided in proportion to the population size of the towns, as reported in the last census of 1998. One union council from each of the towns was randomly selected and further divided into blocks, each comprising 50 households. Proportionate numbers of blocks were chosen at random from the list of all the given blocks. Finally, 10 eligible households with one or more persons aged 17–21 years were chosen using systematic sampling (every fifth household from a total of 50 households in the selected block). In all, 165 blocks were chosen and, by taking 10 households from each block, the desired sample size was reached.

If there was more than one eligible person in the household, one was randomly chosen. Equal numbers of males and females were selected. There were 10–15 per cent refusals in some of the blocks. This was taken into account by moving to the next household until the desired sample was obtained.

A questionnaire based on existing validated instruments from national surveys of reproductive health of youth in Pakistan was used [18,19], but it was supplemented with context-specific items to assess the knowledge and awareness of behaviours related to HIV/AIDS. The questionnaire also contained items on socio-demographic and psychosocial conditions. The same questionnaire was used for males and females and was pre-tested prior to the data collection.

The data were collected by a team of male and female interviewers of about the same age as the male and female respondents in order to facilitate communication. The interviews were conducted at their homes, ensuring that no other person could overhear the conversation. Six interviewers (three males and three females) were trained at a two-day workshop (classroom discussion sessions) to ensure a full understanding of the instrument and its purpose. The training was conducted by the principal investigator, supported by a field manager and a field supervisor, who together developed the instruction manual for the interviewer training.

### Dependent variable

Two open-ended questions assessed knowledge of HIV/AIDS; one on the modes of spread phrased 'What are the various modes by which HIV/AIDS is being spread' and the other on preventive strategies phrased 'Name a few strategies using which an individual can prevent himself/herself from getting infected with HIV/AIDS'. The responses were assessed as 'correct' or 'incorrect' by the researchers. Each of the two variables was then coded into four categories, i.e. 'poor knowledge' (no correct answer), 'some knowledge' (1 correct answer), 'good knowledge' (2 correct answers) and 'very good knowledge' (3 or more correct answers). A composite variable based on the two items was created and defined at three levels, i.e. 'poor knowledge' (both variables had only incorrect responses), 'some knowledge' (exactly one variable had at least one correct response) and 'good knowledge' (both variables had at least one correct response). For multivariate analyses, the composite variable was dichotomised into 'poor knowledge' as opposed to 'some knowledge' and 'good knowledge' taken together.

### Independent variables

Socio-economic status (SES) was based on the possession of assets by the households. Initially, an average current price in the market was assigned to the various assets and scores were developed on the basis of their values. Socio-economic status was dichotomised at the median (Rs.20, 000/- equal to US$340/-) into medium to high as opposed to low SES. As total monthly income was reported by only 23 per cent of the study subjects, it could not be used.

Age was classified into two groups; 17–18 and 19–21 years of age. Educational level was based on the number of years of education and dichotomised into those with ≥ 6 years of schooling and < 6 years. Enrolment at school/college was classified as enrolled or not enrolled. Working status was defined as those in paid employment/voluntary work as opposed to those not in paid employment/voluntary work, including students. Marital status was defined as ever married or unmarried. Residential status was classified as permanent or migrant; those ethnic groups who were native to the city of Karachi at the time of independence (1947), including the migrants from India, were classified as permanent. Family type was dichotomised into 'nuclear families' (living with their children only) as opposed to 'extended families' (grandparents, grandchildren or in-laws also living as household members).

### Statistical procedures

Cronbach's alpha coefficient was used to measure the internal consistency of the items defining the dependent variable, knowledge of HIV/AIDS; it was 0.75. Odds ratios were used to estimate bivariate associations between socio-demographic and psychosocial factors and knowledge of HIV/AIDS. Multiple logistic regression analyses further identified different tentative models of association and possible confounding factors. Only those variables that displayed statistically significant associations in the bivariate analyses were entered stepwise. Marital status was only marginally significant in the bivariate analysis for females, but it was included in the logistic regression models due to its inherent importance. All the analyses were performed separately for males and females.

The data were double entered into Epi Info version 6.04d [17]. Ten per cent of the questionnaires were checked randomly to look for any error in the data entry. SPSS version 15.0 [20] was used for the data analysis.

### Ethical considerations

Ethical approval for the study was obtained from the Aga Khan University Ethical Review Committee (AKU-ERC). Informed consent was obtained both from the parents and from the respondents. Due to the sensitivity of reproductive health matters among youth in Pakistan's social context, great care was taken in training the interviewers not to cause any argument or controversy while approaching the respondents.

## Results

Average life circumstances in the study population differed for males and females. More females belonged to the migrant group and more females also lived in an extended family setting, had a lower socio-economic status and reported not being in paid employment as compared to the males (Table 1). Only 7.2 per cent of the participants were married and they were mainly females. Access to TV was higher among females (93.7 per cent) than males (86.4 per cent), while more males (25.7 per cent) than females (18.1 per cent) had access to computers (Table 1).

**Table 1:** Socio-demographic and psychosocial characteristics, n = 1,650 (males = 826, females = 824).

Characteristic	All		Males		Females	
	n	%	n	%	n	%
**Age (years)**						
17–18	685	41.5	339	41.0	346	42.0
19–21	965	58.5	487	59.0	478	58.0

**Educational level**						
Illiterate	174	10.5	71	8.6	103	12.5
Can read a newspaper	3	0.2	0	0.0	3	0.4
Can read a newspaper and write a letter	1	0.1	0	0.0	1	0.1
1–5 years of schooling	106	6.4	46	5.6	60	7.3
6–10 years of schooling	998	60.5	537	65.0	461	55.9
11–12 years of school/college	317	19.2	152	18.4	165	20.0
> 12 years of school/college	51	3.1	20	2.4	31	3.8

**Years of schooling**						
< 6	284	17.2	117	14.2	167	20.2
≥ 6	1366	82.8	709	85.8	657	79.8

**Current enrolment in school/college**						
Yes	811	49.2	473	57.3	338	41.0
No	839	50.8	353	42.7	486	59.0

**Current working status**						
Student	824	49.9	476	57.6	348	42.2
Staying at home/Housewife	310	18.8	0	0.0	310	37.6
Unemployed	253	15.3	153	18.5	100	12.1
Paid job	189	11.5	155	18.8	34	4.1
Self employed	44	2.7	36	4.4	8	1.0
Volunteer work	30	1.8	6	0.7	24	2.9

**Occupational status**						
Non working (including student)	1387	84.1	629	76.1	758	92.0
Working	263	15.9	197	23.9	66	8.0

**Marital status**						
Unmarried	1532	92.8	823	99.6	709	86.0
Ever married	118	7.2	3	0.4	115	14.0

**Residential status**						
Permanent	1131	68.5	604	73.1	527	64.0
Migrant	519	31.5	222	26.9	297	36.0

**Family setup**						
Extended	333	20.2	48	5.8	285	34.6
Nuclear	1317	79.8	778	94.2	539	65.4

**Socio Economic Status.**						
High to middle	697	42.2	364	44.1	333	40.4
Low	953	57.8	462	55.9	491	59.6

**Television in the home**						
Yes	1486	90.1	714	86.4	772	93.7
No	164	9.9	112	13.6	52	6.3

**Computer in the home**						
Yes	361	21.9	212	25.7	149	18.1
No	1289	78.1	614	74.3	675	81.9

### 'Not heard' of HIV/AIDS

Of the total population of 1,650 adolescents, 390 reported that they had not heard of HIV/AIDS, i.e. 221 (26.8 per cent) males and 169 (20.5 per cent) females.

Due to the relatively high proportion of young people who were ignorant on HIV, analyses were made to compare those who had not heard of HIV/AIDS (n = 390) with those who had some knowledge (n = 1260). The males who had not heard of HIV/AIDS were less well educated in that 76.0 per cent had ≥ 6 years of school/college, as compared to 89.4 per cent (p < 0.001) among those reporting a knowledge of HIV, and had less access to computers (13.1 per cent vs 30.2 percent; p < 0.001). Similarly, the females who had not heard of HIV/AIDS were less well educated (68.6 per cent as compared to 82.6 per cent; p < 0.001), more frequently belonged to a family with low socio-economic status (76.9 per cent vs 55.1 per cent; p < 0.001) and fewer were married (12.4 per cent vs 14.4 per cent; p 0.52) as compared to those reporting a knowledge of HIV/AIDS.

### Knowledge and awareness of HIV

Varied responses were noted for knowledge and awareness among the 1,260 reporting knowledge of HIV. A screening question inquiring about whether or not HIV/AIDS is curable revealed that only 32 per cent of the males but 55 per cent of the females gave a correct 'no' response to this question. However, almost twice as many females (28.1 per cent) as males (15.4 per cent) reported that they did not know how the HIV/AIDS virus is spread (Table 2).

**Table 2:** Suggestions for modes of spread of HIVAIDS among males and females, n = 1,260 (males = 605, females = 655)*

FACTORS	All		Males		Females	
	n	%	n	%	n	%
Sexual contact	719	57.1	428	70.7	291	44.4
Re-used syringe	306	24.3	48	7.9	258	39.4
HIV/AIDS-positive blood infusion	195	15.5	44	7.3	151	23.1
Used blade/razor	56	4.4	19	3.1	37	5.6
HIV/AIDS-positive mother to newborn transmission	40	3.2	10	1.7	30	4.6
Nose/ear piercing and grafting names on skin	6	0.5	0	0	6	0.9
Improperly cleaned operative instruments	7	0.6	0	0	7	1.1
Eating with HIV/AIDS-positive patient	14	1.1	0	0	14	2.1
Coughing from HIV/AIDS-positive patient	3	0.2	1	0.2	2	0.3
Talking to HIV/AIDS-positive patient	3	0.2	1	0.2	2	0.3
Shaking hands with HIV/AIDS-positive patient	9	0.7	1	0.2	8	1.2
Uncleanliness	37	2.9	0	0	37	5.6
Other	27	2.1	3	0.6	24	3.7
Don't know	277	22	93	15.4	184	28.1

* Multiple responses

Of the males, 70.7 per cent reported sexual contact as a mode of spread, as did 44.4 per cent of the females. A difference was noted for 'used syringe' and 'HIV/AIDS-positive blood infusion', where substantially more females than males believed these contributed to the spread of HIV/AIDS (Table 2). Nevertheless, misconceptions were also identified, such as talking, shaking hands, coughing or eating with an HIV/AIDS-infected person. These misconceptions were more common among the females (Table 2).

Regarding ways of preventing the further spread of HIV/AIDS, 34.7 per cent of the females and 41.3 per cent of the males reported having no knowledge of preventive measures (Table 3). Further, 41.3 per cent of the males and 32.4 per cent of the females reported avoidance of sex as a preventive measure, but, when the females and males who also mentioned avoiding 'extramarital sex' were added together, the difference was smaller (Table 3). Here, too, substantially more females than males appeared to be aware of blood contamination as a mode of spread (Table 3).

**Table 3:** Suggestions for preventive strategies regarding HIV/AIDS among males and females, n = 1,260 (males = 605, females = 655).*

FACTORS	All		Males		Females	
	n	%	n	%	n	%
Avoiding sex	462	36.7	250	41.3	212	32.4
Using new syringe	159	12.6	38	6.3	121	18.5
Avoiding extramarital sexual contacts	131	10.4	41	6.8	90	13.7
Using screened blood	92	7.3	2	0.3	90	13.7
Avoiding used blade/razor for shaving	46	3.7	29	4.8	17	2.6
Faithful only to marital partner	22	1.7	7	1.2	15	2.3
Avoiding use of items of HIV/AIDS patient	8	0.6	1	0.2	7	1.1
Avoiding grafting names on skin	3	0.2	0	0	3	0.5
Other	25	2.3	5	1.3	18	3.1
Don't know	477	37.9	250	41.3	227	34.7

* Multiple responses

**Figure 1:**
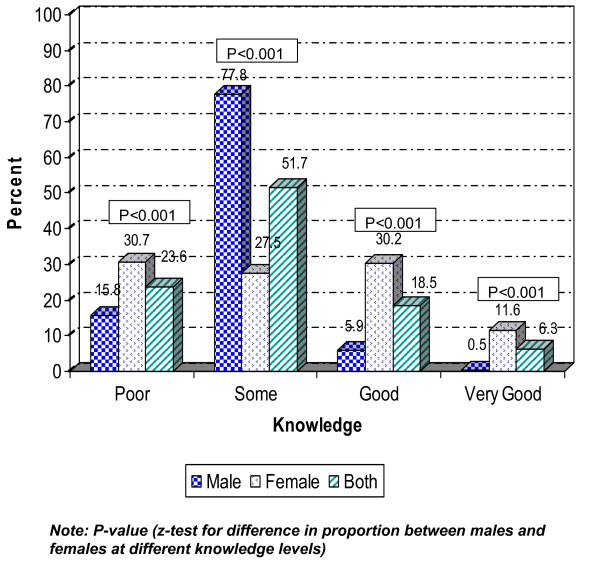
**Knowledge level of modes of spread of HIV/AIDS by sex, n = 1,260 (males = 605, females = 655)**.

**Figure 2:**
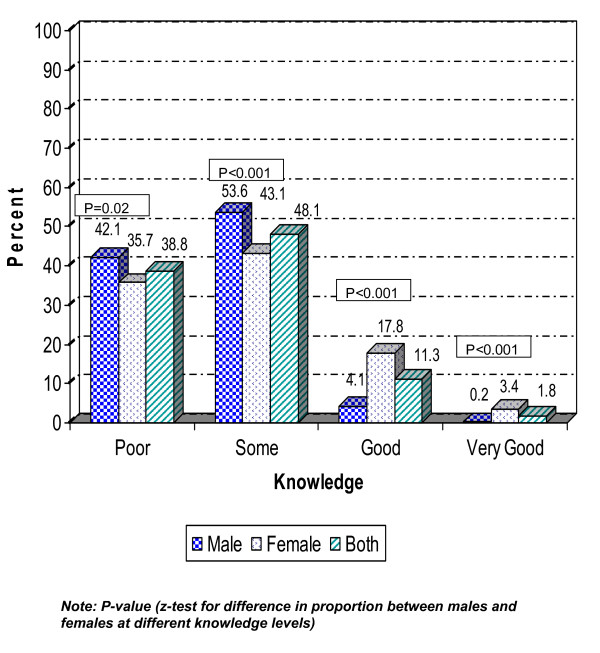
**Knowledge level of preventive strategies for HIV/AIDS by sex, n = 1,260 (males = 605, females = 655)**.

In the next step, the level of knowledge was categorised as very good, good, some or poor knowledge, depending on of the number of the suggested measures that were classified as correct. Almost twice as many females as males were categorised as having poor knowledge of the modes of spread for HIV/AIDS, but, on the other hand, females considerably outnumbered males in the 'very good' knowledge category. For preventive strategies, the females were more knowledgeable than the males and statistically significant differences were found between females and males at all knowledge levels, as presented in Figure 1 and 2.

**Figure 3:**
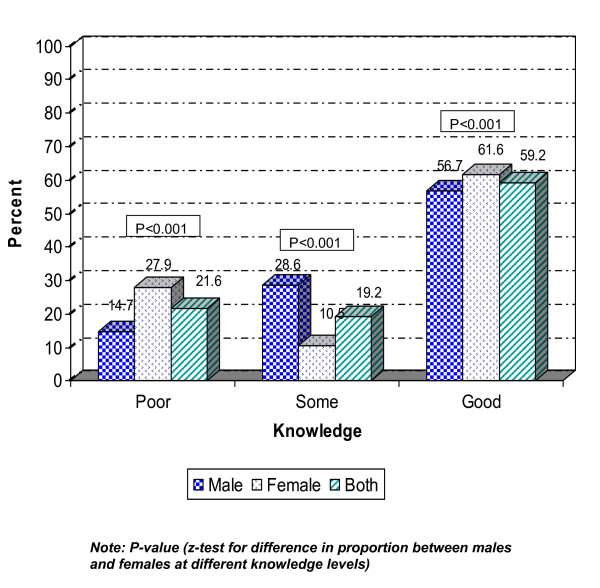
**Summarised knowledge variable and distribution by sex, n = 1,260 (males = 605, females = 655)**.

Figure 3, which shows the summarised variable, reveals that more females than males had a 'good knowledge' (61.6 and 56.7 per cent respectively), while substantially more females than males also displayed a 'poor knowledge' of HIV/AIDS (27.9 and 14.7 per cent respectively).

Risk factor analysis using the summarised knowledge variable revealed that, in the case of males, young age (17–18 years) (crude odds ratio, COR = 2.16; 95% confidence interval, CI, 1.37, 3.40), less than six years of schooling (COR = 2.84; 1.57, 5.14), migrant residential status (COR = 1.68; 1.03, 2.72) and no computer in the household (COR = 2.0; 1.15, 3.52) were associated with poor knowledge (Table 4). Young age (17–18 years) was also significantly associated with a poor knowledge of HIV/AIDS among the females (COR = 1.72; 1.22, 2.44), as was a lack of enrolment at school/college (COR = 1.43; 1.01, 2.04) and low socio-economic status (COR = 1.71; 1.20, 2.43), (Table 4).

**Table 4:** Association between socio-demographic and psychosocial factors and knowledge of HIV/AIDS.

	*Males (n = 605)*			*Females (n = 655)*		
***Socio-demographic***	***All***	***Poor knowledge***	***Crude OR***	***All***	***Poor knowledge***	***Crude OR***
***Factors***	***N***	***n %***	***95% CI***	***N***	***n %***	***95% CI***

**Age group (years)**						

19–21	382	42 (11.0%)	1	392	92 (23.5%)	1

17–18	223	47 (21.1%)	2.16 (1.37, 3.40)	263	91 (34.6%)	1.72 (1.22, 2.44)

**Years of schooling**						

≥ 6	541	70 (12.9%)	1	541	144 (26.6%)	1

< 6	64	19 (29.7%)	2.84 (1.57, 5.14)	114	39 (34.2%)	1.43 (0.93, 2.21)

**Enrolment (school/college)**						

Yes	364	51 (14.0%)	1	273	65 (23.8%)	1

No	241	38 (15.8%)	1.15 (0.73, 1.81)	382	118 (30.9%)	1.43 (1.01, 2.04)

**Working status**						

Not working (including students)	468	64 (13.7)	1	602	168 (27.9)	1

Working	137	25 (18.2)	1.41 (0.85, 2.34)	53	15 (28.3)	1.02 (0.55, 1.90

**Marital status**						

Ever married	3	0 (0.0)	-	94	19 (20.2%)	1

Unmarried	602	89 (14.8)	-	561	164 (29.2%)	1.63 (0.96, 2.78)

**Residential status**						

Permanent	455	59 (13.0%)	1	418	126 (30.1%)	1

Migrant	150	30 (20.0%)	1.68 (1.03, 2.72)	237	57 (24.1%)	0.73 (0.51, 1.06)

**Family type**						

Extended	35	6 (17.1%)	1	243	61 (25.1%)	1

Nuclear	570	83 (14.6%)	0.82 (0.33, 2.04)	412	122 (29.6%)	1.25 (0.88, 1.80)

**Socio-economic status**						

High to middle	301	42 (14.0%)	1	294	65 (22.1%)	1

Low	304	47 (15.5%)	1.13 (0.72, 1.77)	361	118 (32.7%)	1.71 (1.20, 2.43)

**Own TV**						

Yes	546	78 (14.3%)	1	610	170 (27.9%)	1

No	59	11 (18.6%)	1.38 (0.68, 2.76)	45	13 (28.9%)	1.05 (0.54, 2.05)

**Own computer**						

Yes	183	17 (9.3%)	1	122	36 (29.5%)	1

No	422	72 (17.1%)	2.00 (1.15, 3.52)	533	147 (27.6%)	0.91 (0.59, 1.40)

Odds ratios and 95% confidence intervals, n = 1,260.

The multivariate analyses were performed separately for males and females to explore possible confounding factors and chains of associations. For the males, all the variables apart from migrant residential status remained statistically significant in the final model (Table 5). For the females, young age, low SES and no enrolment at school were identified as risk factors for a poor knowledge of HIV/AIDS, but this also applied to being unmarried (AOR = 1.85; 1.05, 3.26), although marital status was marginally significant in the bivariate analysis (Table 4). There were no correlations exceeding 0.4 between independent variables in any of the models (Cramer's V).

**Table 5:** Association between selected socio-demographic factors and poor knowledge of HIV/AIDS among males and females, presented as *adjusted OR *and *confidence intervals *(95% CI), final models, n = 1,260.

*Socio-demographic factors*	*Males (n = 605)*	*Females (n = 655)*
**Age (years)**		
19–21	1	1
17–18	2.20 (1.38, 3.49)	1.74 (1.22, 2.50)

**Years of schooling**		
≥ 6	1	
< 6	2.46 (1.29, 4.68)	

**Own computer**		
Yes	1	
No	1.88 (1.06, 3.34)	

**Residential status**		
Permanent	1	
Migrant	1.23 (0.72, 2.10)	

**Socio-economic status**		
High to middle		1
Low		1.54 (1.06, 2.22)

**Enrolment school/college**		
Yes		1
No		1.61 (1.09, 2.39)

**Marital status**		
Ever married		1
Unmarried		1.85 (1.05, 3.26)

## Discussion

This study found serious gaps in knowledge and awareness among young Pakistanis, aged 17 to 21, regarding the modes of spread and ways of preventing the further transmission of the HIV/AIDS virus. It is most worrying that, when those in the 'poor knowledge' category were added to those who had not heard about HIV/AIDS, 38 per cent of the males and 43 per cent of the females from the total population in this study were ignorant about HIV. These young people were mainly found in lower socio-economic strata.

A substantial percentage of males and of females who had heard about HIV/AIDS were, however, not knowledgeable about HIV being an incurable disease, the way HIV is spread or ways of preventing the further spread. Among those with "some knowledge" of HIV/AIDS, males performed better than females, even though slightly more females than males were found in the "good knowledge" category. The risk factors analysis supported the well-known fact that young age and level of education are important, as is socio-economic status. Nevertheless, computer access contributed to acquiring knowledge about HIV/AIDS for the males, while this was not the case for the females. Unmarried females were found to be less well informed than married females.

### Methodological considerations

As this was a cross-sectional study, it is by definition impossible to assess the direction of some of the associations. Poor knowledge about HIV/AIDS is however most probably due to a lack of enrolment at school/college or low educational achievement, as there is a time sequence. The lack of a computer in a household may lead to poor knowledge about HIV/AIDS and, similarly, young age is associated with less knowledge, which is logical.

Three items were designed to assess HIV/AIDS knowledge. One was used only as a pointer (whether HIV is curable), while the other two were open ended and only these were selected for further analysis (modes of spread and preventive strategies) as these items required the respondent to take a stand to formulate an answer, as opposed to when pre-set responses are used. This procedure was judged to sufficiently reflect knowledge and awareness.

If the participant gave at least one accurate response, it was counted as 'correct'. This somewhat liberal attitude might, lead to an overestimation of the level of knowledge and awareness among adolescents, which means that our figures relating to 'poor knowledge' are not inflated.

A multi-stage cluster sampling technique was used to identify the participants, based on stringent criteria. The sample was collected from all over Karachi and randomness was ensured at each step in order to obtain a representative population. However, there were 10–15 per cent refusals in some of the blocks in the higher socio-economic status areas. This was taken into account by moving to an adjacent household and there is no reason to believe that this changed the representativeness of the study population in terms of socio-economic status, as blocks are fairly homogeneous in this respect.

The interviewers were well versed with the questionnaire and they strictly followed the instruction manual. Furthermore, the field manager scrutinised each form at the field site for completion and good quality of data. However, due to strong religious and cultural beliefs, only questions phrased in a general sense could be asked and issues such as the individuals' own sexual experiences could not be discussed. Despite these constraints, we believe this study was able to identify knowledge and awareness among the young people to benefit the design of appropriate interventions.

As concerns generalisability of our findings, we consider them applicable to urban populations throughout Pakistan, yet the situation is somewhat different in northern parts of the country with in general a lower level of education in the population.

### Comparing results with other studies

In this study population, more males than females reported not having heard about HIV/AIDS (76 per cent). A national survey reported that about 91 per cent of young people had heard of HIV/AIDS in an urban setting [18]. This slightly higher figure was due to the age bracket, which was somewhat higher, up to 24 years.

A study from Chile, investigating knowledge of HIV/AIDS among 15–19 year olds found a different pattern in that no significant differences were found in terms of knowledge of preventive practices related to HIV/AIDS according to either gender or educational level [21]. A study from the United Arab Emirates investigating HIV knowledge among first-year university students found that serious misconceptions existed and women were less knowledgeable than men [2].

It was somewhat surprising to discover that more females than males responded in the affirmative when asked 'have you heard of HIV/AIDS'. This could be explained by the fact that females are more exposed to TV and radio broadcast campaigns about HIV/AIDS, as they spend more time in the home. These campaigns may have some effect in conveying the message [18], but, in this study, access to TV did not indicate any association with deeper knowledge of HIV/AIDS. Having a computer in the home did, however, contribute to improved knowledge among males but not among females. This indicates that, even though there are computers in the homes, the females are not expected to use them. It appears that females are mainly dependent on relatives and peers for knowledge and information about HIV/AIDS, which need further exploration.

When it came to the mode of spread and preventive strategies for HIV/AIDS, a high percentage knew about the source through which the infection could spread, which is quite similar to the findings reported in a national survey [18]. In our study, more males than females named sexual contact as the major mode of spread, while more females than males mentioned used syringes. This finding could be explained by the fact that females are more reluctant to mention sexual matters, while males can talk openly to a stranger (interviewer) about it as a result of the prevailing gender practices in this culture.

When knowledge about HIV/AIDS was assessed on the basis of a composite variable, more females were distributed at either extreme, i.e. more females had a 'poor knowledge' but, at the same time, more females had a 'good knowledge' compared with the males. This dispersion is most probably explained by the vulnerable situation most females have to face with lower school attendance and higher levels of illiteracy among females as compared to males. At the same time, some females, mainly from families with a higher socio-economic status, are given the opportunity to enter into higher education and they perform well.

In some studies conducted in neighbouring India, it was revealed that female adolescents were less knowledgeable about HIV/AIDS compared with male adolescents, while the males reported significantly greater exposure to HIV/AIDS education compared with the females [22]. Moreover, there was a substantial gap in the knowledge of and attitudes towards HIV/AIDS and other sexually transmitted diseases, but males performed somewhat better than females [23]. Nevertheless having good knowledge about HIV/AIDS does not necessarily translate into healthy behaviours [24].

It was further found that the risk factor profile differed somewhat between the males and the females. These differences are due to women and men living different lives in Pakistan, mainly due to gender disparities. This is specifically evident in terms of access to education [19]. In addition, investment in primary education is higher for males than for females [25]. A recent demographic survey showed that education is positively related to knowledge of HIV/AIDS [26], which supports the findings in this study.

## Conclusion

Our findings suggest that there is a huge need to educate young adults and equip them with sufficient information and skills about HIV/AIDS and to further support them to adopt healthy behaviours to prevent a more widespread epidemic in the general population. This is a matter not only of educating the young people, it also involves a change of attitudes and beliefs to take the final step also to change behaviour. This study is able to show that there are huge gaps in knowledge and awareness and to build a healthy nation the other steps towards changing behaviours also needs attention from policy makers, health staff, the school system and families.

The school system needs to assume responsibility for life skills education of which sexual and reproductive health issues constitute a main part. Though, the young adults who are outside the formal educational system also need to be informed. Community leaders, youth clubs and peers are key people at grass root level.

Taboos surrounding the public discussion of sexuality remain a key constraint to preventive activities. Parents often feel ill equipped to talk to their children, even though they may be young people's preferred source of information, but also school teachers have been mentioned in this respect. Political leaders need to address young people's need for information, education and services, investing in national gender awareness programmes to support the health and development of young people.

However, as the prevalence of HIV/AIDS is still comparatively low, the epidemic has not yet enforced a general discussion on the importance of a well-informed younger generation. More qualitative research, addressing the perceptions of young people themselves, is needed.

The findings from this study will provide input for policy formulation and programme design, addressing adolescents' awareness of HIV/AIDS.

## Competing interests

The authors declare that they have no competing interests.

## Authors' contributions

SFH was the principal investigator and was involved in all steps up to manuscript writing, EJ contributed in the logistics while GK helped in analysis and critical review of the manuscript. All authors have read and approved the final manuscript.

## Pre-publication history

The pre-publication history for this paper can be accessed here:

http://www.biomedcentral.com/1471-2334/9/38/prepub
